# Early morning physical activity is associated with healthier white matter microstructure and happier children: the ActiveBrains project

**DOI:** 10.1007/s00787-023-02197-6

**Published:** 2023-04-14

**Authors:** Irene Esteban-Cornejo, Inmaculada Lara-Jimenez, Maria Rodriguez-Ayllon, Juan Verdejo-Roman, Andres Catena, Kirk I. Erickson, Francisco B. Ortega

**Affiliations:** 1 Department of Physical Education and Sports, Faculty of Sport Sciences, Sport and Health University Research Institute (iMUDS), University of Granada, Carretera de Alfacar s/n, 18071 Granada, Spain; 2 Centro de Investigación Biomédica en Red Fisiopatología de la Obesidad y Nutrición (CIBERobn), Instituto de Salud Carlos III, 28029 Madrid, Spain; 3 Department of Epidemiology, Erasmus MC University Medical Center Rotterdam, Rotterdam, The Netherlands; 4 Department of Personality, Assessment and Psychological Treatment, Mind, Brain and Behavior Research Center (CIMCYC), University of Granada, Granada, Spain; 5 Department of Experimental Psychology, University of Granada, Granada, Spain; 6 Department of Psychology, Center for the Neural Basis of Cognition, University of Pittsburgh, Pittsburgh, PA USA; 7 AdventHealth Research Institute, Neuroscience, Orlando, FL USA; 8 Faculty of Sport and Health Sciences, University of Jyväskylä, Jyvaskyla, Finland; 9 Instituto de Investigación Biosanitaria ibs.GRANADA, Granada, Spain

**Keywords:** Neurodevelopment, Psychological health, White matter integrity, Physical activity, Diet, Sleep, Childhood, Obesity

## Abstract

The background of this study is to examine the associations of individual and combined early morning patterns (i.e., active commuting to school, physical activity before school, having breakfast and good sleep) with white matter microstructure (WMM) and, whether the associated white mater microstructure outcomes were related to mental health outcomes in children with overweight or obesity. 103 children with overweight or obesity (10.0 ± 1.1 years old, 42 girls) from the ActiveBrains project participated in this cross-sectional study. Early morning patterns and mental health indicators (i.e., self-esteem, optimism, positive and negative affect, stress, depression and anxiety) were self-reported by the children using validated questionnaires. WMM was assessed by magnetic resonance imaging using diffusion tensor imaging. When examined independently, early morning patterns were not related with WMM (all *P* > 0.05). However, the combination of early morning patterns was related with WMM (*P* < 0.05). Specifically, physically active early morning patterns (i.e., active commuting to school and physical activity before school) were associated with global fractional anisotropy (FA) (*β* = 0.298, *P* = 0.013) and global radial diffusivity (RD) (*β* = − 0.272, *P* = 0.021), as well as with tract-specific FA (*β* = 0.314, *P* = 0.004) and RD (*β* = − 0.234, *P* = 0.032) in the superior longitudinal fasciculus (SLF). Furthermore, combined physically active early morning pattern-associated global (i.e., FA and RD) and tract-specific (i.e., FA and RD in the SLF) WMM indicators were positively associated with happiness (*β* absolute value range from 0.252 to 0.298, all *P* < 0.05). A combination of physically active early morning patterns may positively relate to white matter microstructure in children with overweight or obesity, and, in turn, happiness.

**Supplementary Information:**

The online version contains supplementary material available at 10.1007/s00787-023-02197-6.

## Introduction

Childhood is a critical period for neurodevelopment [1]. In particular, the brain undergoes significant changes in white matter structure [2]. The structure of white matter is responsible for providing fast and efficient transmission of information between brain areas into structural networks to support cognition and mental health [3, 4]. Indeed, white matter microstructure was related to general and specific psychopathology symptoms (e.g., internalizing and externalizing problems) in children [5]. Emerging studies have considered white matter as one brain feature susceptible for modification by lifestyle behaviors (e.g., physical activity, sleep or diet) during childhood [6–9], which in turn may be related to childhood mental health outcomes [5].

Specifically, we have previously shown that physical activity was related to enhanced white matter microstructure in the present sample of children with overweight and obesity [7]. In addition, we observed in a population-based study conducted in normal-weight children that total physical activity (i.e., outdoor playing and sport participation) was associated with improved white matter microstructure indicators [8]. Importantly, new paradigms based on chronotype suggest that physically active early morning behaviors may confer additional health benefits, such as dementia risk [10]. Likewise, irrespective of total physical activity, morning physical activity has been associated with better cardiometabolic health outcomes [11, 12] and lower risks of incident cardiovascular diseases [13]; this highlights the potential importance of chronoactivity mainly in physical physiological health, which might be extended to brain health. However, the previous studies have not examined the association of early active morning patterns with white matter microstructure. The only study examining active commuting, included both active commuting to (during mornings) and from (during afternoon) school, and found no associations with white matter indicators [8]. In addition, other early morning factors, such as having breakfast or good sleep, may also influence white matter microstructure [6, 9, 14]. For example, sleep disturbances were negatively associated with white matter microstructure in preadolescents [9]. In addition, having bread (compared with rice) for breakfast was associated with greater white matter volume in children [14].

Beyond the influence of individual behaviors on white matter, an approach that combines multiple behaviors, has been broadly recommended based on the multifactorial etiology of chronic diseases and behavioral outcomes [15]; in turn, considering the cumulative, or combined, effects of each behavior may have a larger impact on health outcomes than any single factor [16]. As far as we know, there has not been a previously published study that has examined the association between individual and combined early morning patterns on white matter microstructure in children with overweight or obesity. Specifically, obesity is associated with alterations in white matter properties as well as development of psychiatric disorders as compared to normal-weight individuals [17–19]. For example, obese children have shown worse white matter microstructure in the cerebellar peduncles and higher risk of both anxiety and depression than their normal-weight peers [20, 21]. Therefore, there is a clear need for studies that examine different early morning patterns and their associations with white matter microstructure in overweight/obese children, as well as their coupled influence on mental health.

The aim of the present study was to examine the associations of individual and combined early morning patterns (i.e., active commuting to school, physical activity before school, having breakfast and good sleep) with white matter microstructure and, whether the associated white matter microstructure outcomes were related to mental health in children with overweight or obesity.

## Methods

### Participants

The present cross-sectional study was developed within the Active Brains Project framework (http://profith.ugr.es/). A detailed description of the study design and methods has been published elsewhere [22]. A total of 110 children with overweight and obesity (8–11 years old) from Granada (Spain) were recruited and assessed from 2014 to 2016. Children were categorized as overweight and obesity grade I/II/III according to age- and sex-specific BMI cut points [23, 24]. Of these, 103 (42 girls) were included in the present analyses. The ActiveBrains study was approved by the Review Committee for Research Involving Human Subjects at the University of Granada and was registered in ClinicalTrials.gov (Identifier: NCT02295072).

### Early morning patterns

Early morning patterns included both physically active behaviors (i.e., physical activity before school and active commuting to school) and non- physically active behaviors (i.e., having breakfast and good sleep). *Physical activity before school* and *active commuting to school* were assessed by the Youth Activity Profile-Spain (YAP-S) questionnaire. The YAP-S is a cross-translated and adapted version of the original YAP (available at: http://profith.ugr.es/yap?lang=en). The original YAP was calibrated through a series of studies by Saint Maurice et al. [25, 26]. The YAP-S questionnaire showed adequate reliability for activity at school and out-of-school [weighted kappa coefficient –*K*– = 0.66–0.76; the intraclass correlation coefficient (ICC) = 0.83–0.86] in children [27]. Participants were asked on “how many days” during the last week: (i) they performed physical activity before school (from 6.00 a.m. to 9.00 a.m.) for at least 10 min (not including walk or bike to school) and (ii) they walked or cycled to school; answer ranging from 0 to 5 days. Each answer was classified into two categories as never (0 days) vs. some days (1 to 5 days).

*Having breakfast* was evaluated by an item from the KIDMED Questionnaire [28]. This questionnaire consists of 16 yes/no questions regarding the Mediterranean diet, and includes an item for skipping breakfast. This answer was inverted as not having breakfast vs. having breakfast. Although, a classical validation study has not been performed due to the structure of the KIDMED questionnaire; there is evidence that higher scoring of the KIDMED index is associated with expected patterns of food and nutrient intakes indicating good dietary quality, demonstrating its construct validity [29, 30], and has been extensively used in youth Spanish population [31–33].

*Good sleep* was defined as combining quantity and quality of sleep. Sleep quantity was evaluated by the Paediatric Sleep Questionnaire (PSQ) and sleep quality was evaluated by the Child Health Utility 9D Questionnaire (CHU9D). The PSQ was completed by the parents according to their child’s usual sleep habits and includes two items on the normal time to go to bed and the time of day for the child to get out of bed during a weekday. The Spanish version of the PSQ showed good reliability for usual sleep habits (*K* > 0.50) [34]. Sleep duration was calculated and recoded as meeting the sleep recommendation for children by the National Sleep Foundation (9–11 h) vs. not meeting the sleep recommendation [35]. The CHU9D consists of 9 items to assess the child’s functioning across several domains including sleep problems. Participants rated their response using a 5-point Likert type scale, from 1 = “no sleep problems” to 5 = “almost couldn’t sleep”; answers were categorized as no sleep problems [1] vs. others [2–5]. The Cronbach alpha for the CHU9D was 0.781, indicating an acceptable level of internal consistency [36]. The label of *good sleep* was defined based on two categories: either meeting the sleep recommendation and not experiencing sleep problems vs. others (i.e., not meeting the recommendations or the presence of sleep disturbances).

### Magnetic resonance imaging (MRI) procedure

#### Imaging acquisition

MRI data were collected with a 3.0 Tesla Siemens Magnetom Tim Trio scanner (Medical Solutions, Erlangen, Germany) [7]. Diffusion tensor imaging (DTI) data were acquired with an Echo Planar Imaging (EPI) sequence following the parameters: echo time (TE) = 90 ms, trace of the tensor (TR) = 3300 ms, field of view (FOV) = 230 mm × 230 mm, flip angle = 90, matrix = 128 × 128, slice thickness = 4 mm, number of slices = 25 and voxel resolution = 1.8 × 1.8 × 4 mm^3^. One volume without diffusion weighting (*b* = 0 s/mm^2^) and 30 volumes with diffusion weighting (*b* = 1000 s/mm^2^) were collected [7]. We use fractional anisotropy (FA), mean diffusivity (MD), radial diffusivity (RD), and axial diffusivity (AD) as derived DTI metrics. FA shows the degree to which water diffuses preferentially along one axis and increases during child development and to be lower in neurological and psychiatric diseases. MD is a scalar describing the average diffusion in all directions, with higher levels indicating relatively unimpeded diffusion (i.e., negatively correlated with FA). RD measures water diffusion perpendicular to the axonal wall. AD represents the diffusivity of water in the direction parallel to the fiber bundles in anisotropic tissue, such as white matter. High values of FA and AD, and low values of MD and RD are shown as indicators of healthier white matter microstructure during childhood [37].

#### Imaging preprocessing

Image preprocessing was carried out through the Functional MRI of the Brain Software Library (FSL) [38]. First, we corrected for eddy current-induced distortions and participant movement with the 6.0.1 version of eddy correction (https://fsl.fmrib.ox.ac.uk/fsl/fslwiki/eddy). Next, outliers were replaced by non-parametric predictions by the Gaussian Process. To account for rotations applied to the imaging data during motion correction, the resulting transformation matrices were used to rotate the diffusion gradient direction table. Non-brain tissue was removed using the FSL Brain Extraction Tool. Finally, the diffusion tensor was fit, and common scalar maps (i.e., FA, MD, RD and, AD) were estimated.

#### Probabilistic fiber tractography

Probabilistic fiber tractography was performed using the fully automated FSL plugin, “AutoPtx” (https://fsl.fmrib.ox.ac.uk/fsl/fslwiki/AutoPtx). Diffusion data were processed with the Bayesian Estimation of Diffusion Parameters Obtained using Sampling Techniques (BEDPOSTx), considering two fiber orientations at each voxel [39]. Then, for each participant, the FSL nonlinear registration tool (FNIRT) was used to align the FA map to the FMRIB-58 FA template image. The inverse of the nonlinear warp field was calculated and applied to a series of predetermined target, seed, exclusion, and termination masks produced by the AutoPtx plugin [40].

Probabilistic fiber tracking was carried out with the FSL Probtrackx module using the supplied tract-specific masks (i.e., target, seed, etc.) that were deformed to the native diffusion image space of each subject. The resulting path distributions were normalized to a scale from 0 to 1 using the total number of successful seed-to-target attempts and low-probability voxels likely related to noise were removed as a result of the established threshold.

White matter tract segmentation was accomplished by thresholding the normalized tract density images based on previously established values [cingulate gyrus part of cingulum (CGC): 0.01, corticospinal tract (CST): 0.005, inferior longitudinal fasciculus (ILF): 0.005, superior longitudinal fasciculus (SLF): 0.001, uncinate fasciculus (UNC): 0.01, forceps major (FMA): 0.005, forceps minor (FMI): 0.01] [40]. Next, average FA, MD, RD, and AD values were calculated for each tract. Connectivity distributions were estimated for seven large fiber bundles (i.e., CGC, CST, ILF, SLF, UNC, FMA, and FMI) selected based on previous reports [4, 40]. To assess whether exposures were related to global measures of white matter integrity (i.e., global FA, MD, RD, and AD), selected tracts were combined into a single factor “Global Factor”. The global factor was calculated by averaging all tracts and weighting this average by the size (volume) of the tracts (in the neuroimaging literature, in particular in cortical morphology studies, it is a common practice to ensure that small regions do not contribute equally as larger regions) [8, 9]. Left and right hemispheres were averaged for the global and individual tracts’ DTI metrics.

#### Image quality assurance

Image quality assurance was performed following the previous procedures used in children [8]. Image quality was classified using a 4-point scale with values 1 = “excellent”, 2 = “minor”, 3 = “moderate”, and 4 = “severe”. Datasets which had insufficient quality (i.e., moderate and severe) for statistical analyses were excluded (*n* = 2). The sum-of-squares error maps from the tensor estimation were computed and visually inspected for structured noise in FSL. First, the native space FA map registration was examined to ensure that images were all correctly aligned to the template (masks were properly mapped to native space). Second, all tracts were visualized to ensure rigorous path reconstruction. Raw image quality and probabilistic tractography data were visually examined by a rater blind to clinical data, and a second rater checked it in case of ambiguity [4].

### Mental health indicators

Mental health indicators were divided into two groups: psychological well-being (i.e., happiness, self-esteem, optimism and positive affect) and psychological ill-being (i.e., stress, depression anxiety and negative affect) [41].

*Happiness* was measured with the 4-item Subjective Happiness Scale (SHS). The subjects responded to 4 questions with answers ranging from 1 to 7. The score was obtained from the sum of the 3 first items with values ranging from 3 (low happiness) to 21 (high happiness). The Spanish version of SHS showed an adequate internal consistency (Cronbach’s alpha ranged from 0.79 to 0.94), appropriate test–retest reliability (Pearson’s *r* ranged from 0.55 to 0.90) and convergent validity (Pearson’s *r* ranged from 0.52 to 0.72) [42].

*Self-esteem* was evaluated by the Rosenberg Self-Esteem Scale (RSE). The test includes 10 items measuring positive and negative feelings. The final score is a measure of global self-worth. Higher scores indicate higher self-esteem. The RSE is a reliable (Cronbach’s alpha = 0.86) and valid (Pearson’s *r* = 0.41) self-report to assess self-esteem in children [43].

*Dispositional optimism* was measured with the Life Orientation Test-Revised (LOT-R). This test contains 10 items from pessimism to optimism, and the final score was calculated by summing the 6 items that assessed optimism. LOT-R is a valid, reliable and useful self-report measure to evaluate optimism in children (Cronbach’s alpha = 0.78 and Pearson’s *r* ranged for test–retest reliability from 0.56 to 0.79) [44].

*Stress* was assessed by The Children’s Daily Stress Inventory (CDSI). The CDSI measures the prevalence of stressful events in four areas: school, friends, health and family. The answers are dichotomous with yes/no items. The result of the final sum indicates a high or low level of stress. The inventory has good reliability (Pearson’s *r* = 0.78) and validity (Cronbach’s alpha = 0.70) in primary school students from Spain [45].

*Depression* was evaluated by the Children’s Depression Inventory (CDI). It measures symptoms related to dysthymic disorder or depression in children. The 27 items are grouped into five factor areas: interpersonal problems, negative mood, anhedonia, ineffectiveness and negative self-esteem. The final score was calculated summing values from 0 (low depression level) to 54 (high depression level). The Cronbach alpha for this test has reported to be 0.84 for males and 0.87 for females [46].

*Childhood trait anxiety* was assessed by the State-Trait Anxiety Inventory for Children (STAIC-T). The STAI measures tension, apprehension, worry and nervousness, but is normally used as a global anxiety measure. The inventory has 20 items with scores categorized from 1 to 3 (almost never-often). A high score is indicative of a high trait anxiety level. STAIC-T is widely used, reliable and extensively validated (Cronbach alpha = 0.94) [47].

*Positive and negative affect* was evaluated by The Positive and Negative Affect Schedule for Children (PANAS-C). The PANAS-C has 20 items with answers ranging from 1 to 3. Positive affect was calculated by summing 10 items, and negative affect was obtained from the sum of 10 items; higher scores reflect higher positive and negative affect. Prior data have shown a Cronbach alpha from 0.87 to 0.90 for the positive affect subscale and 0.87–0.94 for the negative affect subscale [48].

### Covariates

Sex, peak height velocity (PHV, year) and parent education university level were included as covariates based on the previous studies from the ActiveBrains project [49–52]. PHV is a common indicator of maturity (i.e., biological age) in children and adolescents [53]. PHV was obtained from anthropometric variables (weight, height and/or seated height) using Moore’s equations [54]. Parent education, a proxy measure of socioeconomic status, was defined by the highest completed education and divided into 3 groups ranging from 0 (neither), 1 (one of parent) or 2 (both parents). In sensitivity analyses, additional covariates were included [body mass index (BMI), other physical activity, watching TV and Mediterranean Diet Index]. Body weight and height were obtained with participants barefoot and wearing underclothes. Weight was measured with an electronic scale (SECA 861, Hamburg, Germany), and height (cm) with a stadiometer (SECA 225, Hamburg, Germany). Both measurements were performed twice and averages were used. BMI was expressed as kg/m^2^. Physical activity and watching TV were assessed by the Youth Activity Profile-Spain (YAP-S) questionnaire [27] and Mediterranean Diet Index by the KIDMED questionnaire [28].

### Statistical analysis

All analyses were performed using the Statistical Package for Social Sciences (IBM SPSS Statistics for Windows, version 23.0) and the level of significance was set to 0.05. Characteristics of the study sample are presented as means and standard deviations (SD) or percentages, after checking for normality using Kolmogorov–Smirnov test. Multiple linear regression analyses adjusting for basic confounders (i.e., sex, PHV and parental education) are presented as standardized β coefficients (95% confidence interval, *P*). First, we studied the associations between individual early morning patterns (i.e., active commuting to school, physical activity before school, having breakfast and good sleep) as independent variables and global white matter microstructure indicators (i.e., global FA, global MD, global RD and global AD) as dependent variables. Second, we categorized the data as physically active early morning patterns (i.e., sum of active commuting to school and physical activity before school, and operationalized as 0 physically active patterns, 1 pattern or 2 patterns) and non-physically active early morning patterns (i.e., sum of having breakfast and good sleep, and operationalized as 0–1 non-physically active patterns or 2 patterns, since only 3 participants had 0 patterns), to examine the associations of combined physically active and non-physically active early morning patterns with global white matter microstructure indicators. Third, to determine whether the relationship with white matter microstructure was global or restricted to a particular set of white matter bundles, associations with white FA, MD, RD, and AD within individual tracts were also tested if exposures showed an association with global DTI metrics.

In addition, a number of sensitivity analyses were run: (i) basic significant models were additionally adjusted for BMI, physical activity, watching TV or Mediterranean diet, (ii) non-physically active early morning pattern models were repeated excluding those with 0 patterns, and (iii) including both physically active and non-physically active early morning patterns in the same regression model; that is, when the physically active early morning pattern variable was modelled as the main exposure, the analysis was also adjusted for the non-physically active early morning pattern variable, and when the non-physically active early morning pattern variable was modelled as the main exposure, the analysis was also adjusted for the physically active early morning pattern variable.

Finally, the associations of global white matter microstructure indicators as independent variables with mental health indicators as dependent variables, adjusting for basic confounders (i.e., sex, PHV and parental education), were performed if early morning patterns showed an association with global DTI metrics. Similarly, associations of FA, MD, RD, and AD within individual tracts with mental health indicators were also tested for those mental health indicators associated with global DTI metrics. Main analyses were corrected for multiple comparisons using the Benjamini–Hochberg method based on each DTI metric (30 comparisons for FA, 14 for MD, 30 for RD and 14 for AD) [55].

## Results

The descriptive characteristics of the study sample are shown in Table 1. A total of 103 children (42 girls, mean age 10 ± 1.1 years) with mean BMI of 26.7 ± 3.6 kg/m^2^ were included in this study. The descriptive values for global and tract-specific white matter microstructure indicators are shown in Table S1.

**Table 1:** Sample characteristics

	*N*	Mean/%	SD
All	103		
Girls, %	42	40.8	
Age (years)	103	10.0	1.1
Peak height velocity (years)	103	− 1.90	1.0
Weight (kg)	103	56.0	11.3
Height (cm)	103	144.2	8.6
Body mass index (kg/m^2^)	103	26.7	3.6
Body mass index status (%)	103		
Overweight	27	26.2	
Obesity type 1	45	43.7	
Obesity type 2	20	19.4	
Obesity type 3	11	10.7	
Parental education university level (%)	103		
Neither parent	66	64.1	
One parent	19	18.4	
Both parents	18	17.5	

Values are expressed as means ± standard deviations, unless otherwise indicated

### Individual early morning patterns and global white matter microstructure

The associations of individual early morning patterns with global white matter microstructure indicators are shown in Table 2. Overall, no associations were found between individual morning patterns and global white matter microstructure indicators (all *P* > 0.05).

**Table 2:** Associations of individual early morning patterns with global white matter microstructure indicators

		GLOBAL FA		GLOBAL MD		GLOBAL RD		GLOBAL AD	
	*N*	*β* (95% CI)	*P*	*β* (95% CI)	*P*	*β* (95% CI)	*P*	*β* (95% CI)	*P*
Active commuting to school									
No	44	Ref		Ref		Ref		Ref	
Yes	45	0.213 (− 0.003, 0.430)	0.054	− 0.165 (− 0.355, 0.047)	0.123	− 0.196 (− 0.392, 0.016)	0.071	− 0.061 (− 0.265, 0.143)	0.570
Physical activity before school									
No	59	Ref		Ref		Ref		Ref	
Yes	30	0.140 (− 0.071, 0.351)	0.191	− 0.132 (− 0.308, 0.066)	0.201	− 0.146 (− 0.333, 0.062)	0.164	− 0.072 (− 0.311, 0.144)	0.488
Having breakfast									
No	10	Ref		Ref		Ref		Ref	
Yes	65	− 0.094 (− 0.325, 0.137)	0.421	0.103 (− 0.137, 0.342)	0.376	0.101 (− 0.130, 0.317)	0.384	0.081 (− 0.163, 0.342)	0.490
Good sleep^†^									
No	24	Ref		Ref		Ref		Ref	
Yes	61	0.046 (− 0.172, 0.264)	0.677	− 0.015 (− 0.218, 0.196)	0.885	− 0.029 (− 0.343, 0.257)	0.791	0.014 − 0.193, 0.232)	0.892

*β* values are standardized regression coefficients. Analyses were adjusted for sex, peak height velocity (year) and parent education university level (neither/one/both)

*FA* Fractional anisotropy, *MD* mean diffusivity, *RD* Radial diffusivity, *AD* Axial diffusivity, *Ref.* reference

^†^Good sleep was calculated as those meeting the sleep recommendation (9–11 h) and reported not sleep problems

### Combined physically active and non-physically active early morning patterns with global white microstructure

Physically active morning patterns were associated with greater global FA (*β* = 0.298, *P* = 0.013) and lesser global RD (*β* = − 0.272, *P* = 0.021), but not associated with global MD (*P* = 0.054) or global AD (*P* = 0.524). No association was found between non-physically active early morning patterns and global indicators (all *P* > 0.05) (Table 3). In sensitivity analyses, the association between non-physically active early morning patterns with global white matter microstructure indicators was studied excluding those with 0 patterns (*n* = 3) and results were similar (Table S2). Results remained similar when basic significant models were additionally adjusted for BMI, physical activity, watching TV, or Mediterranean diet (Table S3). In addition, when we included both physically active and non-physically active early morning patterns in the same regression model, results were virtually the same (data no shown).

**Table 3:** Associations of combined physically active and non-physically active early morning patterns with global white matter microstructure indicators

		GLOBAL FA		GLOBAL MD		GLOBAL RD		GLOBAL AD	
	*N*	*β* (95% CI)	*P*	*β* (95% CI)	*P*	*β* (95% CI)	*P*	*β* (95% CI)	*P*
Physically active patterns^†^									
0 pattern	28	Ref		Ref		Ref		Ref	
1 pattern	47	0.108 (− 0.128, 0.344)	0.365	− 0.171 (− 0.391, 0.049)	0.145	− 0.155 (− 0.377, 0.067)	0.187	− 0.155 (− 0.399, 0.089)	0.191
2 patterns	14	0.298 (0.064, 0.532)	0.013*	− 0.225 (− 0.433, 0.004)	0.054	− 0.272 (− 0.509, − 0.034)	0.021*	− 0.075 (− 0.283, 0.149)	0.524
Non-physically active patterns^‡^									
0–1 pattern	26	Ref		Ref		Ref		Ref	
2 patterns	44	0.081 (− 0.157, 0.320)	0.497	0.016 (− 0.225, 0.248)	0.891	− 0.022 (− 0.285, 0.220)	0.854	0.091 (− 0.160, 0.365)	0.451

*β* values are standardized regression coefficients. Analyses were adjusted for sex, peak height velocity (year) and parent education university level (neither/one/both). Statistically significant values are shown in bold (*P* < 0.05)

*FA* Fractional anisotropy, *MD* mean diffusivity, *RD* Radial diffusivity, *AD* Axial diffusivity, *Ref.* reference

*Statistically significant values surpassed multiple comparisons using the Benjamini and Hochberg method

^†^Combined physically active patterns were calculated as the sum of performing active commuting to school and physical activity before school

^‡^Combined non-physically active patterns were calculated as the sum of having breakfast and good sleep

### Combined physically active early morning patterns with tract-specific white matter microstructure

Associations of combined physically active early morning patterns with tract-specific white matter microstructure indicators are shown in Table 4. Compared to those with 0 physically active early morning patterns, those with 2 physically active early morning patterns had higher FA (*β* = 0.314, *P* = 0.004) and lower RD (*β* = − 0.234, *P* = 0.032) in the SLF. Results were similar when separately run the associations for left and right SLF FA (left SLF: *β* = 0.367, *P* = 0.002; right SLF: *β* = 0.374, *P* = 0.002) and RD (left SLF: *β* =− 0.327, *P* = 0.006; right SLF: *β* =− 0.361, *P* = 0.003) indicators. Results were virtually the same when basic significant models were additionally adjusted for BMI, physical activity, watching TV or Mediterranean diet (Table S4). No other associations were found with white matter metrics (i.e., FA, and RD) in any other tract (*P* > 0.05).

**Table 4:** Associations of combined physically active early morning patterns with tract-specific white matter microstructure indicators

		FA		RD	
	*N*	*β* (95% CI)	*P*	*β* (95% CI)	*P*
Cingulate gyrus part of cingulum					
0 pattern	28	Ref		Ref	
1 pattern	47	− 0.007 (− 0.249, 0.235)	0.956	− 0.003 (− 0.246, 0.246)	0.982
2 patterns	14	0.031 (− 0.210, 0.271)	0.801	− 0.095 (− 0.355, 0.150)	0.433
Corticospinal tract					
0 pattern	35	Ref		Ref	
1 pattern	53	0.072 (− 0.144, 0.288)	0.512	− 0.076 (− 0.266, 0.133)	0.485
2 patterns	15	0.157 (− 0.059, 0.373)	0.153	− 0.186 (− 0.386, 0.029)	0.090
Inferior longitudinal fasciculus					
0 pattern	35	Ref		Ref	
1 pattern	53	0.034 (− 0.187, 0.255)	0.760	− 0.029 (− 0.205, 0.161)	0.786
2 patterns	15	0.150 (− 0.071, 0.372)	0.181	− 0.193 (− 0.410, 0,024)	0.077
Superior longitudinal fasciculus					
0 pattern	35	Ref		Ref	
1 pattern	53	− 0.001 (− 0.209, 0.206)	0.989	− 0.010 (− 0.216, 0.197)	0.923
2 patterns	15	0.314 (0.104, 0.523)	0.004*	− 0.234 (− 0.441, − 0.028)	**0.032**
Uncinate fasciculus					
0 pattern	35	Ref		Ref	
1 pattern	53	0.002 (− 0.223, 0.227)	0.985	0.020 (− 0.243, 0.284)	0.857
2 patterns	15	0.039 (− 0.186, 0.264)	0.730	− 0.083 (− 0.309, 0.142)	0.461
Forceps major					
0 pattern	35	Ref		Ref	
1 pattern	53	0.047 (− 0.176, 0.269)	0.679	− 0.167 (− 0.392, 0.050)	0.130
2 patterns	15	0.163 (− 0.060, 0.386)	0.151	− 0.117 (− 0.339, 0.099)	0.288
Forceps minor					
0 pattern	35	Ref		Ref	
1 pattern	53	− 0.009 (− 0.234, 0.215)	0.934	0.014 − 0.313, 0.342)	0.901
2 patterns	15	0.007 (− 0.218, 0.232)	0.951	− 0.020 (− 0.231, 0.198)	0.862

*β* values are standardized regression coefficients. Analyses were adjusted for sex, peak height velocity (year) and parent education university level (neither/one/both). Statistically significant values are shown in bold (*P* < 0.05). Combined physically active patterns were calculated as the sum of performing active commuting to school and physical activity before school

*FA* Fractional anisotropy, *RD* Radial diffusivity, *Ref.* reference

*Missing values by preprocessing

*Statistically significant values surpassed multiple comparisons using the Benjamini and Hochberg method

### Combined physically active early morning pattern-associated global and tract-specific white matter indicators with mental health

**Fig. 1:**
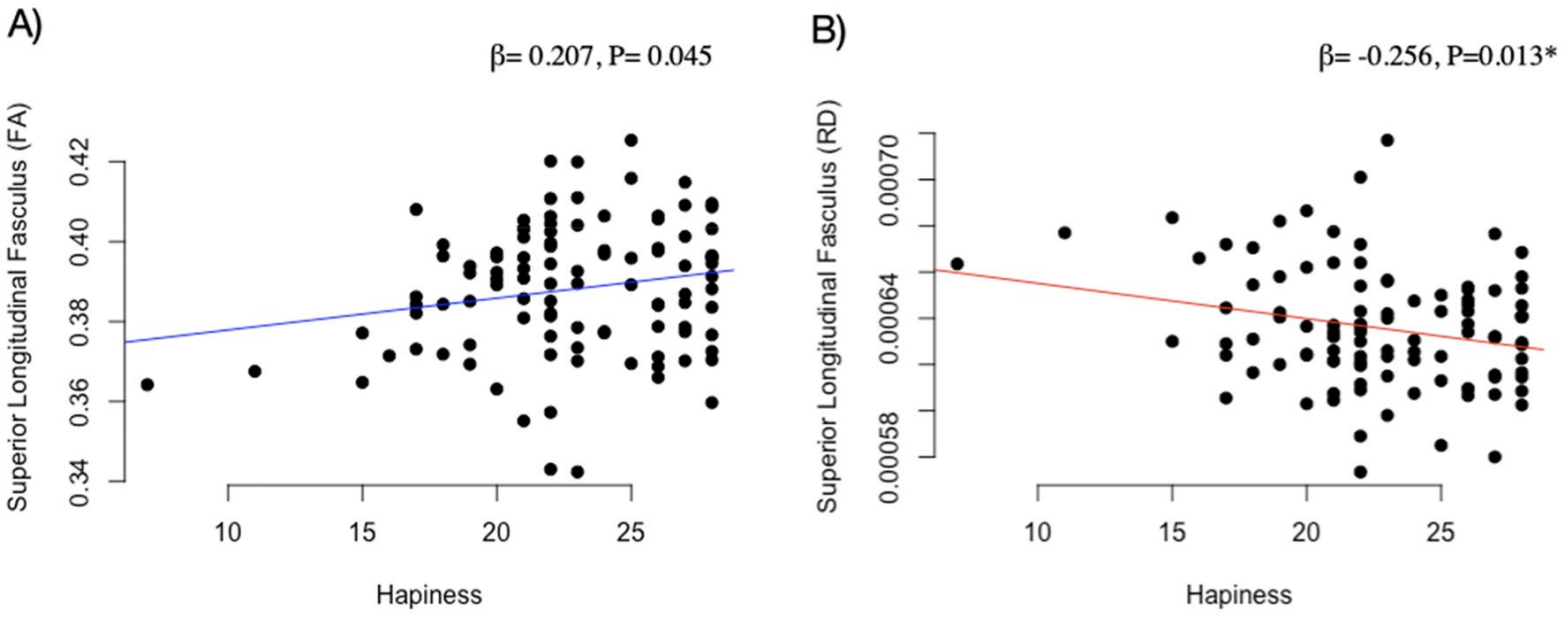
Associations of tract-specific white matter microstructure indicators with mental health indicators. Panel **A** Fractional anisotropy (FA); Panel **B** Radial Diffusivity (RD). *β* values are standardized regression coefficients. Analyses were adjusted for sex, peak height velocity (year), and parent education university level (neither/one/both). Superior longitudinal fasciculus was selected, since it was the tract previously associated with active morning patterns. *Statistically significant values surpassed multiple comparisons using the Benjamini and Hochberg method

The associations of mental health indicators with that of the combined physically active early morning patterns that were associated with global white matter indicators are shown in Table 5. Global FA was positively associated with happiness (*β* = 0.252, *P* = 0.022). In addition, global RD (*β* = − 0.298, *P* = 0.008) was negatively associated with happiness. No other associations were found between global white matter microstructure indicators and mental health outcomes (*P* > 0.05). Finally, FA (*β* = 0.207, *P* = 0.045) and RD (*β* = − 0.256, *P* = 0.013) in the SLF was related to happiness (Fig. 1).

**Table 5:** Associations of combined physically active early morning patterns-associated global white matter indicators with mental health indicators

		Global FA		Global RD	
	*N*	*β* (95% CI)	*P*	*β* (95% CI)	*P*
Positive psychological health					
Happiness (SHS)	89	0.252 (0.037, 0.467)	0.022*	− 0.298 (− 0.515, − 0.081)	0.008*
Self-esteem (RSE)	88	0.053 (− 0.158, 0.264)	0.620	− 0.080 (− 0.295, 0.135)	0.461
Optimism (LOT-R)	88	0.142 (− 0.079, 0.363)	0.206	− 0.177 (− 0.401, 0.047)	0.120
Positive affect (PANAS-C)	86	− 0.142 (− 0.363, 0.079)	0.206	0.096 (− 0.131, 0.322)	0.404
Negative psychological health					
Stress (CDSI)	87	− 0.017 (− 0.233, 0.198)	0.873	− 0.010 (− 0.229, 0.209)	0.927
Depression (CDI)	88	0.085 (− 0.130, 0.301)	0.433	− 0.064 (− 0.285, 0.157)	0.568
Anxiety (STAIC-R)	85	0.079 (− 0.119, 0.278)	0.428	− 0.123 (− 0.327, 0.080)	0.232
Negative affect (PANAS-C)	84	− 0.029 (− 0.253, 0.196)	0.800	0.113 (− 0.114, 0.340)	0.324

*β* values are standardized regression coefficients. Analyses were adjusted for sex, peak height velocity (year) and parent education university level (neither/one/both). Statistically significant values are shown in bold (*P* < 0.05)

*FA* Fractional anisotropy, *RD* Radial diffusivity, *SHS* Subjective Happiness Scale, *RSE* The Rosenberg Self-Esteem Scale, *LOT-R* Life Orientation Test-Revised, *PANAS-C* Positive and Negative Affect Scale for Children, *CDSI* Children’s Daily Stress Inventory, *CDI* Children Depression Inventory, *STAIC-R* State-Trait Anxiety Inventory for Children

*Statistically significant values surpassed multiple comparisons using the Benjamini and Hochberg method

## Discussion

Individual early morning patterns or combined non-physically active early morning patterns were not associated with white matter microstructure indicators. However, our findings suggest that a combination of physically active early morning activities, particularly active commuting to school and physical activity before school, is related to global and SLF tract-specific white matter indicators (FA, RD); and in turn, those white matter indicators are related to happiness. Therefore, these novel results suggest synergistic effects of physically active early morning patterns on white matter microstructure, coupled with better happiness in children with overweight or obesity.

Previous studies examined the associations between individual lifestyle behaviors (e.g., physical activity, sleep or diet) and white matter microstructure in children [6–9]. For example, a larger study in normal-weight children found that total physical activity (i.e., outdoor playing and sport participation) was associated with improved white matter indicators (i.e., higher global FA and lower global MD); however, active commuting, including both active commuting to (during mornings) and from (during afternoon) school, was not associated with white matter [8]. Another study using the present sample of children with overweight or obesity showed that total physical activity was related to greater white matter microstructure (i.e., global FA) [9]. In addition, non-physically active behaviors (having breakfast or good sleep) may also influence white matter [6, 9, 14]. Specifically, breakfast staple type was differentially associated with white matter volume in children [14] and sleep disturbances were related to less white matter microstructural integrity (i.e., lower FA) [9]. In contrast, we found that individual early morning patterns (i.e., active commuting to school, physical activity before school, having breakfast and good sleep) were not associated with white matter microstructure in children with overweight or obesity. These discrepancies between studies may be due to: (i) characteristics of the sample (i.e., normal-weight children vs. children with overweight/obesity), since overweight/obese children may adhere to unhealthier behaviors and show differential white matter development compared with their normal-weight peers [56–58], and (ii) methodological differences, since early morning patterns were assessed using different self-reported questionaries across studies. Thus, future studies are needed to understand the individual associations of early morning patterns and white matter microstructure using standardized methodologies and comparing the whole range of body mass index distribution in children.

Importantly, we found that the combination of non-physically active early morning patterns (i.e., having breakfast and good sleep) was not significantly related to white matter indicators; however, the combination of physically active early morning patterns was associated with white matter microstructure. Specifically, compared to those having 0 active patterns, those performing both patterns, active commuting to school and physical activity before school, had greater white matter microstructure. Importantly, these results were independent of other factors not occurring in the morning, such as other physical activity, watching TV, BMI or Mediterranean diet index. Therefore, these results highlight the possible importance of engaging physical activity in the morning, and walking or cycling to school for white matter microstructure in childhood. Although identifying time-dependent biological mechanisms is beyond the scope of this study, we can speculate about several potential reasons underlying the morning activity-white matter associations. Biological processes (e.g., metabolism, hormone production, immunity, or behavior) are orchestrated by circadian clocks, and temporal gating of those processes is essential for maintaining homeostasis. Particularly, timing of physical activity can entrain circadian control of systemic energy homeostasis and behavioral activity [59, 60]. For example, a study in mice found that physical activity timing is a critical factor to amplify the benefits of exercise on metabolic pathways in skeletal muscle and systemic energy homeostasis; specifically decreasing muscle and blood glucose levels only after early morning activity [59]. These physiological adaptations, might in turn, influence brain tissue, by secreted exerkines crossing the blood–brain barrier [61]. Indeed, a study in mice found that kynurenine was increased only after morning physical activity [60]; kynurenine directly targets important neurotransmitter receptors and affects redox processes, and thus likely influences brain physiology [62]. Likewise, ketones such as beta-hydroxybutyrate were remarkably increased by morning physical activity, indicating a greater reliance on fatty acid oxidation and increased buffering against metabolic stress, which may exert neuroprotective effects [63]. Finally, hypothalamic neurotransmitters serotonin, dopamine, and its catabolite homovanillate only increase by morning physical activity [61]. Thus, based on animal models, a time-dependent impact of physical activity on the human brain seems to be biologically plausible, albeit it is important to highlight that exercise physiology and circadian rhythms are highly variable in humans and animals. Future longitudinal studies and clinical trials in humans are needed to confirm the synergistic- and early morning-specific effects of active behaviors on white matter microstructure, as well as its potential underlying mechanisms.

Another interesting finding was that in addition to the associations with global white matter microstructure, there was a tract-specific association between a combination of physically active early morning patterns and white matter microstructure in the SLF. The SLF connects parietal, temporal and occipital regions to the frontal lobe. This tract is involved in supporting different functions including processing of visual spatial information, decision-making and emotional regulation [64–66]. In particular, studies in children have associated the SLF with several different processes and functions [5, 67, 68]. For instance, Bruckert et al. [67] found an association between SLF in both hemispheres with later reading in children and Brandes-Aitken et al. [68] observed an association with both visuomotor control and cognitive control. Moreover, a large population-based study of 10-year-old children showed that improved white matter microstructure was associated with lower general psychopathology, which in turn was related to happiness [5]. Interestingly, in the present study, we found that both global and tract-specific (i.e., SLF) FA and RD were related to happiness, but not with any other mental health indicator, suggesting a specific role of white matter of the SLF with happiness during childhood. In this line, while no previous studies in children have shown a direct white matter–happiness association, some recent investigations in adults examined this hypothesis [69, 70]. For example, a study in 417 healthy Japanese adults found that FA of several tracts, including SLF, was related with happiness assessed also by the SHS questionnaire [69]. Additionally, a meta-analysis including 37 studies found disruptions in the SLF as the most common white matter alteration in adults suffering from psychiatric emotional conditions such as bipolar disorder, social anxiety disorder, major depressive disorder, post-traumatic stress disorder and obsessive–compulsive disorder [71]. Hence, the SLF, although classically associated with spatial functions [72], may have an emotional subcomponent. However, future longitudinal studies with larger sample sizes should examine the global and tract-specific influence of white matter microstructure on mental health indicators in children.

Finally, several limitations require discussion. First, the cross-sectional design does not allow inferences about causality to any of the associated factors. Second, the sample with overweight and obesity limits the generalizability of our findings to the entire range of the BMI distribution. Third, the voxel size was a nonisotropic 4-mm-section voxel (1.8 × 1.8 × 4 mm^3^). Therefore, FA could be underestimated in regions containing crossing fibers (i.e., SLF). On the other hand, the FA measured in regions without crossing fibers (i.e., CST) is not prone to underestimation. Finally, our study relied on self-reported, while validated, measures to assess early morning patterns. On the other hand, the study has several strengths, including the relatively large sample with neuroimaging data, the analyses of both global and tract-specific white matter indicators, and the assessment of a broad mental health battery.

## Conclusion

Our results suggest that individual early morning patterns were not associated with white matter microstructure in children with overweight or obesity. In addition, while combined non-physically active early morning patterns may not be related to white matter indicators, the combination of the two physically active early morning patterns (i.e., active commuting and physical activity before school) may positively relate to global and SLF tract-specific white matter indicators (FA, RD), and in turn, happiness. Yet, these results need to be confirmed in randomized controlled trials.

From a public health perspective, implementing an early morning exercise intervention may generate healthier brains and happier children. Educational and health institutions should target before physical activity school programs as well as promote strategies to walk or cycle to school during childhood to improve brain and mental health.

### Supplementary Information

Below is the link to the electronic supplementary material.

Supplementary file1 (DOCX 24 kb)

## Data Availability

The dataset supporting the conclusions of this article is available upon reasonable request, by emailing to the corresponding author.
