# Supplementary material for: Early morning physical activity is associated with healthier white matter microstructure and happier children: the ActiveBrains project
Source: Eur Child Adolesc Psychiatry. 2023 Apr 14;33(3):833–45. doi: 10.1007/s00787-023-02197-6 (PMC10894097; doi:10.1007/s00787-023-02197-6)
Supplement: Supplementary file 1 — Supplementary file1 (DOCX 24 kb) [file 787_2023_2197_MOESM1_ESM.docx]

**Table Supplementary S1** Descriptive values for global and tract-specific white matter microstructure indicators

|  |  | **FA** | **MD** | **RD** | **AD** |
| --- | --- | --- | --- | --- | --- |
|  | N | Mean ± SD | | | |
| **Global** | 89 | 0.4563±0.01466 | 0.0008±0.00002 | 0.0006±0.00002 | 0.0013±0.00002 |
| **Cingulate Gyrus part of Cingulum** | 89 | 0.4135±0.03245 | 0.0008±0.00002 | 0.0006±0.00003 | 0.0012±0.00005 |
| **Corticospinal Tract** | 103 | 0.5328±0.02154 | 0.0008±0.00002 | 0.0005±0.00002 | 0.0013±0.00002 |
| **Inferior Longitudinal Fasciculus** | 103 | 0.4802±0.01986 | 0.0009±0.00003 | 0.0006±0.00003 | 0.0014±0.00003 |
| **Superior Longitudinal Fasciculus** | 103 | 0.3880±0.01623 | 0.0008±0.00002 | 0.0006±0.00003 | 0.0012±0.00002 |
| **Uncinate Fasciculus** | 103 | 0.4108±0.02749 | 0.0008±0.00002 | 0.0006±0.00003 | 0.0013±0.00003 |
| **Forceps Major** | 103 | 0.5581±0.02733 | 0.0009±0.00006 | 0.0006±0.00006 | 0.0016±0.00007 |
| **Forceps Minor** | 103 | 0.6334±0.03783 | 0.0008±0.00004 | 0.0005±0.00005 | 0.0016±0.00007 |

Values are expressed as means ± SD (standard deviations). FA= Fractional anisotropy, MD= mean diffusivity, RD= Radial diffusivity, AD= Axial diffusivity.

## Table Supplementary S2 Associations of combined non-physically active early morning patterns excluding those with 0 patterns (n=3) with global white matter microstructure indicators.

|  | | **GLOBAL FA** | | **GLOBAL MD** | | **GLOBAL RD** | | **GLOBAL AD** | |
| --- | --- | --- | --- | --- | --- | --- | --- | --- | --- |
|  | N | β (95%CI) | P | β(95%CI) | P | β(95%CI) | P | β(95%CI) | P |
| **Non-Physically Active Patterns^‡^** |  |  |  |  |  |  |  |  |  |
| 1 pattern | 23 | Ref. |  | Ref. |  | Ref. |  | Ref. |  |
| 2 patterns | 44 | 0.106 (-0.137, 0.349) | 0.385 | -0.003 (-0.243, 0.243) | 0.981 | -0.046 (-0.324, 0.231) | 0.704 | 0.087 (-0.175, 0.349) | 0.480 |

β values are standardized regression coefficients. Analyses were adjusted for sex, peak height velocity (year) and parent education university level (neither/one/both). Statistically signiﬁcant values are shown in bold (p < 0.05). ‡ Combined non-physically active patterns were calculated as the sum of having breakfast and good sleep. FA= Fractional anisotropy, MD= mean diffusivity, RD= Radial diffusivity, AD= Axial diffusivity, Ref. = reference.

## Table Supplementary S3 Associations of combined physically active early morning patterns with global white matter microstructure indicators adjusting for additional confounders.

|  | | **GLOBAL FA** | | **GLOBAL RD** | |
| --- | --- | --- | --- | --- | --- |
|  | N | β (95%CI) | P | β(95%CI) | P |
| **MODEL 1** |  |  |  |  |  |
| 0 pattern | 28 | Ref. |  | Ref. |  |
| 1 pattern | 47 | 0.108 (-0.128, 0.344) | 0.365 | -0.155 (-0.377, 0.067) | 0.187 |
| 2 patterns | 14 | 0.298 (0.064, 0.532) | **0.013** | -0.272 (-0.509, -0.034) | **0.021** |
| **MODEL 1 + BMI** |  |  |  |  |  |
| 0 pattern | 28 | Ref. |  | Ref. |  |
| 1 pattern | 47 | 0.108 (-0.132, 0.348) | 0.374 | -0.150 (-0.364, 0.086) | 0.211 |
| 2 patterns | 14 | 0.298 (0.061, 0.535) | **0.014** | -0.267 (-0.501, -0.033) | **0.025** |
| **MODEL 1 + PA** |  |  |  |  |  |
| 0 pattern | 28 | Ref. |  | Ref. |  |
| 1 pattern | 47 | 0.098 (-0.146, 0.342) | 0.426 | -0.130 (-0.347, 0.109) | 0.282 |
| 2 patterns | 14 | 0.281 (0.026, 0.536) | **0.031** | -0.228 (-0.472, 0.016) | 0.073 |
| **MODEL 1 + TV** |  |  |  |  |  |
| 0 pattern | 28 | Ref. |  | Ref. |  |
| 1 pattern | 47 | 0.125 (-0.108, 0.357) | 0.288 | -0.169 (-0.434, 0.072) | 0.149 |
| 2 patterns | 14 | 0.261 (0.029, 0.494) | **0.028** | -0.242 (-0.468, -0.011) | **0.040** |
| **MODEL 1 + MDI** |  |  |  |  |  |
| 0 pattern | 25 | Ref. |  | Ref. |  |
| 1 pattern | 40 | 0.156 (-0.078, 0.390) | 0.187 | -0.206 (-0.412, 0.021) | 0.085 |
| 2 patterns | 7 | 0.431 (0.196, 0.666) | **0.001** | -0.419 (-0.648, -0.178) | **0.001** |

β values are standardized regression coefficients. Model 1 was adjusted for sex, peak height velocity (year) and parent education university level (neither/one/both). Statistically signiﬁcant values are shown in bold (p < 0.05). Combined physically active patterns were calculated as the sum of active commuting to school and physical activity before school. BMI= Body Mass Index, PA= Physical Activity at school, TV= Television viewing, MDI= Mediterranean Diet Index, FA= Fractional anisotropy, RD= Radial diffusivity, Ref. = reference.

## Table Supplementary S4 Associations of combined physically active early morning patterns with tract-specific white matter microstructure indicators adjusting for additional confounders.

|  | | **SLF FA** | | **SLF RD** | |
| --- | --- | --- | --- | --- | --- |
|  | N | β (95%CI) | P | β(95%CI) | P |
| **MODEL 1** |  |  |  |  |  |
| 0 pattern | 35 | Ref. |  | Ref. |  |
| 1 pattern | 53 | -0.001 (-0.209, 0.206) | 0.989 | -0.010 (-0.216, 0.197) | 0.923 |
| 2 patterns | 15 | 0.314 (0.104, 0.523) | **0.004** | -0.234 (-0.441, -0.028) | **0.032** |
| **MODEL 1 + BMI** |  |  |  |  |  |
| 0 pattern | 35 | Ref. |  | Ref. |  |
| 1 pattern | 53 | 0.001 (-0.208, 0.210) | 0.992 | -0.012 (-0.236, 0.197) | 0.910 |
| 2 patterns | 15 | 0.315 (0.105, 0.526) | **0.004** | -0.236 (-0.443, -0.028) | **0.032** |
| **MODEL 1 + PA** |  |  |  |  |  |
| 0 pattern | 35 | Ref. |  | Ref. |  |
| 1 pattern | 53 | -0.027 (-0.243, 0.190) | 0.808 | 0.005 (-0.216, 0.216) | 0.968 |
| 2 patterns | 15 | 0.276 (0.051, 0.501) | **0.017** | -0.212 (-0.452, 0.014) | 0.071 |
| **MODEL 1 + TV** |  |  |  |  |  |
| 0 pattern | 35 | Ref. |  | Ref. |  |
| 1 pattern | 53 | 0.000 (-0.216, 0.215) | 0.997 | -0.010 (-0.216, 0.197) | 0.923 |
| 2 patterns | 15 | 0.299 (0.086, 0.513) | **0.007** | -0.234 (-0.455, -0.014) | **0.035** |
| **MODEL 1 + MDI** |  |  |  |  |  |
| 0 pattern | 30 | Ref. |  | Ref. |  |
| 1 pattern | 45 | 0.092 (-0.126, 0.311) | 0.401 | -0.121 (-0.363, 0.121) | 0.290 |
| 2 patterns | 8 | 0.454 (0.238, 0.671) | **< 0.001** | -0.386 (-0.607, -0.155) | **0.001** |

β values are standardized regression coefficients. Model 1 was adjusted for sex, peak height velocity (year) and parent education university level (neither/one/both). Statistically signiﬁcant values are shown in bold (p < 0.05). Combined physically active patterns were calculated as the sum of active commuting to school and physical activity before school. BMI= Body Mass Index, PA= Physical Activity at school, TV= Television viewing, MDI= Mediterranean Diet Index, SLF= Superior Longitudinal Fasciculus, FA= Fractional anisotropy, RD= Radial diffusivity, Ref. = reference.
